# Safety of *Lactiplantibacillus plantarum* K014 in Healthy Adults: A Randomized, Double-Blind, Placebo-Controlled, Parallel-Group Trial

**DOI:** 10.3390/nu18091406

**Published:** 2026-04-29

**Authors:** Kar Shin Goh, Chee Ping Chong, Joo Shun Tan, Rhu Yann Ho, Zhang Jin Ng, Ahmad Zaimi bin Abdul Latiff, Sulosanah Sinnasamy, Mohd Hisyamuddin Seberi

**Affiliations:** 1 Bioprocess Technology Division, School of Industrial Technology, Universiti Sains Malaysia, Pulau Pinang 11800, Malaysia; karshin@student.usm.my (K.S.G.); hrwei17@gmail.com (Z.J.N.); 2 Discipline of Clinical Pharmacy, School of Pharmaceutical Sciences, Universiti Sains Malaysia, Pulau Pinang 11800, Malaysia; 3 Discipline of Social & Administrative Pharmacy, School of Pharmaceutical Sciences, Universiti Sains Malaysia, Pulau Pinang 11800, Malaysia; rhuyann@usm.my; 4 Medical Unit, Wellbeing Centre (Health & Dental), Main Campus, Universiti Sains Malaysia, Pulau Pinang 11800, Malaysia; ahmadzaimi@usm.my (A.Z.b.A.L.); sulosanah@usm.my (S.S.); syamseberi@usm.my (M.H.S.)

**Keywords:** *Lactiplantibacillus plantarum* K014, safety, gut and immune health, healthy adults, Malaysia

## Abstract

Background and aims: *Lactiplantibacillus plantarum* is a widely studied probiotic species with well-documented benefits for gastrointestinal function and immune modulation. However, probiotic effects are strain-specific, and the safety of newly identified strains must be clinically established. *L. plantarum* K014, isolated from traditionally fermented vegetables, has not previously been evaluated in human subjects. This study aimed to evaluate the safety and tolerability of *L. plantarum* K014 in healthy Malaysian adults by assessing its effects on anthropometric measures, hematological indices, liver and renal function, gastrointestinal health, and selected immune-related outcomes, including the incidence and severity of common cold symptoms. Methods: This single-center, randomized, double-blind, placebo-controlled trial was conducted over a 6-month period. Of 304 healthy adults screened, 152 were enrolled and randomized in a 1:1 ratio to receive either *L. plantarum* K014 (≥1 × 10^9^ CFU/day) or placebo (maltodextrin), administered daily in sachet form; 125 participants completed the study. Clinical assessments, including physical examination, anthropometric measurements, and blood analyses, were performed at baseline, Month 4, and Month 6. Gastrointestinal symptoms, stool characteristics, and immune-related outcomes were monitored weekly using structured online questionnaires. Results: *L. plantarum* K014 was well tolerated, with no probiotic-related adverse events reported. No clinically significant changes were observed in body weight, BMI, hematological indices, or renal function in either group. Exploratory analyses indicated that participants receiving *L. plantarum* K014 exhibited statistically significant differences in several liver function markers, as well as lower severity of diarrhea and abdominal pain compared with placebo, though these findings were not prespecified efficacy endpoints and should be interpreted cautiously. Similarly, lower weekly ratings of common cold symptoms interfering with work or study were observed in the probiotic group as an exploratory observation. Conclusions: Daily consumption of *L. plantarum* K014 at a dose of ≥1 × 10^9^ CFU for six months was safe and well tolerated in healthy adults. The absence of adverse effects, together with observed trends toward lower gastrointestinal discomfort and immune-related symptoms, supports the suitability of *L. plantarum* K014 for further investigation in efficacy-driven clinical studies.

## 1. Introduction

*Lactic acid bacilli*, the largest genus of lactic acid bacteria, comprises hundreds of species with ongoing discoveries. These Gram-positive, facultatively anaerobic, acid-tolerant bacteria belong to the phylum *Firmicutes* and thrive in nutrient-rich environments due to strict growth requirements [1]. Owing to their long history of use in food fermentation, *Lactiplantibacillus* species are generally regarded as safe for human consumption. *L. plantarum* has been granted Qualified Presumption of Safety (QPS) status and occurs naturally in fermented foods as probiotics [2]. Nevertheless, probiotic effects are strain-specific, and the widespread use of a species does not negate the need for rigorous safety evaluation of newly identified strains intended for human consumption. Studies report good tolerability of *Lactiplantibacillus* strains across diverse populations, including infants, adults, and the elderly [3,4].

Probiotics are live microorganisms that confer health benefits when consumed in adequate amounts. Among these, *Lactiplantibacillus* strains are extensively studied for their roles in gastrointestinal health and immune modulation. *L. plantarum* strains, widely marketed for their safety and efficacy, have been associated with improved gut and immune function [5], prompting further investigation. The gut microbiota is central to nutrition, immunity, and overall health, and probiotics, particularly *Lactiplantibacillus*, help maintain balance by suppressing pathogens and promoting beneficial microbes [6]. For instance, *L. plantarum* Dad-13 was associated with weight reduction in overweight women [7].

Recent studies also highlight the role of the gut microbiota–immune axis, positioning probiotics as a promising strategy for enhancing immunity. Probiotic intake has been associated with reduced upper respiratory tract infection symptoms and improved immune markers. For example, *L. plantarum* DR7 modulated cytokine profiles in adults by reducing pro-inflammatory cytokines and enhancing anti-inflammatory responses, indicating its potential to balance immune activity and support host defense [8]. In short, probiotics can influence immune cell expression and activate immunoregulatory pathways, contributing to strengthened immune responses.

Nonetheless, certain *Lactiplantibacillus plantarum* strains have shown adverse or non-beneficial effects in clinical studies. In IBS patients, *L. plantarum* MF1298 led to worsened symptom scores and [9] an 81% preference for placebo, indicating an unfavorable effect on symptoms. Likewise, another randomized controlled trial found that multiple *L. plantarum* strains failed to improve intestinal permeability after barrier stress, demonstrating that their effects can be limited and strain-dependent [10].

Given this strain specificity, a newly identified strain, *L. plantarum* K014, isolated from fermented vegetables [11], has not yet been evaluated in human clinical studies. Hence, its safety and probiotic potential in humans remain unknown. This study was designed primarily as a safety clinical trial to assess the tolerability and safety profile of *L. plantarum* K014 in healthy adults. Given the influence of regional dietary habits and cultural factors on health outcomes, this study aims to evaluate the safety and tolerability of *L. plantarum* K014 in healthy adults. The study assessed its effects on anthropometric parameters, hematological profiles, blood glucose, lipid levels, and liver and kidney function as compared to a placebo group. Additionally, the impact of *L. plantarum* K014 on gastrointestinal and immune-related health outcomes was examined using structured online questionnaires to establish a comprehensive safety profile within the Malaysian context.

## 2. Methods

### 2.1. Study Design

**Figure 1:**
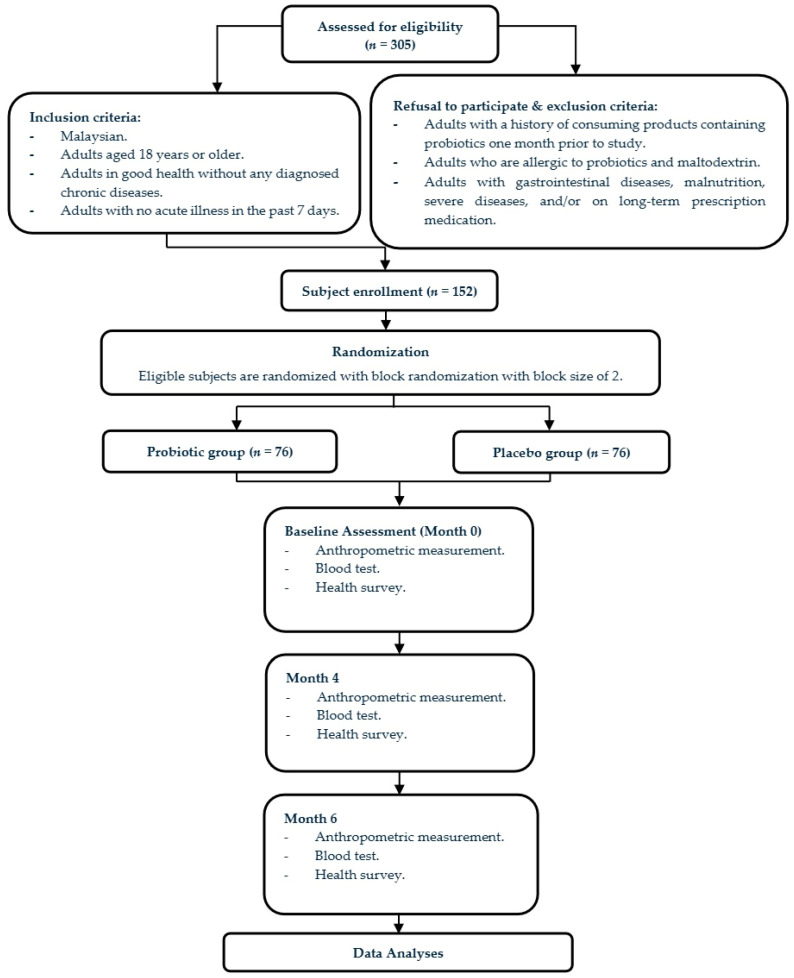
Consort flowchart detailing participants’ recruitment, randomization, allocation, and data collection.

This single-center, randomized, double-blind, placebo-controlled, parallel-group trial was conducted over 24 weeks at Universiti Sains Malaysia (USM), Penang, and was reported in accordance with the CONSORT 2010 guidelines (Table S1). Participants were randomly assigned to receive either *L. plantarum* K014 (probiotic group) or a placebo daily. Assessments were performed at baseline (Month 0), Week 16 (Month 4), and Week 24 (Month 6), including anthropometric measurements and blood tests for hematological parameters, glucose, lipid profile, and liver and kidney function. Gastrointestinal and immune health were monitored through weekly online surveys throughout the study (Figure 1).

### 2.2. Inclusion and Exclusion Criteria

Healthy Malaysian adults aged ≥18 years were recruited. Eligible participants were required to be in good general health and to have an absence of acute illness within seven days prior to enrollment. Exclusion criteria included consumption of probiotics or postbiotics within one month prior to screening; known allergies to probiotics or maltodextrin; a history of gastrointestinal disorders, malnutrition, or severe systemic illness; use of long-term prescription medications; and pregnancy.

### 2.3. Trial Intervention

Participants received either a probiotic formulation or a placebo. The probiotic contained ≥1 × 10^9^ CFU of live *Lactiplantibacillus plantarum* K014 blended with 2 g maltodextrin, while the placebo consisted of 2 g maltodextrin alone. Products were packaged in identical aluminum sachets to ensure blinding. Participants consumed one sachet daily for 24 weeks. Participants were instructed not to mix the sachets with water exceeding 60 °C to maintain probiotic viability. Products were stored below 30 °C, away from direct sunlight, in accordance with manufacturer’s instructions.

### 2.4. Participant Recruitment

Participants were recruited by the study investigators through public advertisements, including promotional posters displayed on the Universiti Sains Malaysia (USM) main campus and announcements disseminated via electronic communication channels. Posters included a QR code linking to an online registration form for interested individuals. Recruitment occurred between April and October 2024, during which 305 individuals registered interest. Following screening against predefined inclusion and exclusion criteria, 152 participants were deemed eligible and enrolled. Prior to enrollment, a trained researcher explained the study objectives, procedures, potential benefits, and risks. To ensure voluntary participation, individuals were given at least 24 h to consider involvement before providing written informed consent. The clinical trial commenced in October 2024 and concluded in March 2025.

### 2.5. Sample Size Determination

The sample size was calculated in accordance with the Malaysian Phase I Clinical Trial Guidelines [12], which recommend enrolling 50–200 healthy volunteers for Phase I safety studies. A minimum of 50 participants per arm was considered appropriate. To accommodate an anticipated 20% dropout rate, 76 participants were recruited per arm, ensuring approximately 60 evaluable subjects per group after attrition.

### 2.6. Sampling and Randomization

A total of 152 participants were enrolled and randomly assigned to either the probiotic or placebo group using simple randomization with a 1:1 allocation ratio. Randomization was performed via GraphPad QuickCalcs (https://www.graphpad.com/quickcalcs/randomize1/, accessed on 25 June 2024) using a computer-generated blocked randomization list with treatment codes. Allocation concealment was maintained by a designated researcher with no participant contact, and the sequence remained inaccessible to the study team until database lock. This was a double-blind trial; both participants and study personnel were unaware of group assignments throughout the study.

### 2.7. Data Collection

At baseline (Month 0), demographic information, anthropometric measurements, and blood samples were collected by trained nurses at the Wellbeing Center (Health and Dental), USM main campus. Participants completed a weekly online health survey throughout the six-month study to monitor gut and immune health. Two follow-up visits were conducted at Month 4 and Month 6, during which anthropometric measurements and blood sampling were repeated.

### 2.8. Safety Assessment

The safety profile of *Lactiplantibacillus plantarum* K014 was evaluated through clinical and laboratory monitoring. Clinical assessments included gastrointestinal and upper respiratory symptoms, as well as any adverse events reported by participants or observed by study staff. Anthropometric measurements and vital signs (height, weight, body mass index [BMI], blood pressure, and heart rate) were recorded at scheduled visits.

Laboratory assessments encompassed hematological, lipid, liver, and renal profiles. Hematological evaluations included red blood cell indices, white blood cell differentials, platelet count, and the inflammatory marker erythrocyte sedimentation rate (ESR). Lipid profile assessments included total cholesterol, triglycerides, high-density lipoprotein (HDL), and low-density lipoprotein (LDL). Liver function was assessed using hepatocellular injury markers (aspartate transaminase [AST] and alanine transaminase [ALT]), a cholestatic marker (alkaline phosphatase [ALP]), synthetic function markers (albumin, total protein, globulin, and albumin/globulin ratio), and an excretory function marker (total bilirubin). Renal function evaluations included waste filtration markers (serum creatinine, blood urea nitrogen [BUN], and uric acid), as well as electrolyte balance indicators (sodium, potassium, and chloride).

### 2.9. Health Survey

The survey component of data collection was conducted using a structured online questionnaire developed based on a validated instrument from the literature [9]. Participants were required to complete the questionnaire once weekly throughout the study period. The questionnaire comprised two main sections: gastrointestinal health (Section A) and immune health (Section B). Section A of the health survey evaluated gastrointestinal health, including symptom frequency, severity, stool characteristics, and diarrhea episodes. Symptom frequency was recorded as the number of days with gastrointestinal symptoms in the past week. Severity was rated on a 6-point Likert scale (0 = no symptoms; 5 = very severe, daily symptoms). Stool consistency was assessed using the Bristol Stool Form Scale, which classifies feces into seven types: Types 1–2 indicate constipation, Types 3–4 are normal, and Types 5–7 reflect diarrhea. Participants reported the number of days each stool type occurred, along with stool color, total weekly bowel movements, and diarrhea frequency. Section B evaluated upper respiratory tract symptoms associated with the common cold. Participants reported the number of days they experienced symptoms during the past week and rated severity using the same 6-point Likert scale applied in Section A (0 = no symptoms; 5 = very severe, daily symptoms). Additionally, interference with daily functioning was assessed using a separate 4-point Likert scale (0 = no interference; 3 = severe interference).

The questionnaire underwent face and content validation by experts from the School of Pharmaceutical Sciences, USM. Item clarity and instructions were refined following pilot testing with five randomly selected healthy volunteers, who were excluded from the final analysis. A forward–backward translation process was employed to develop the Malay version. The original English questionnaire was translated into Malay by a USM language expert and independently back-translated into English by another expert. All versions were reviewed by the expert panel and study investigators to finalize the Malay version for use in the trial.

### 2.10. Adverse Event Reporting

Participants could withdraw at any time, particularly in the event of an adverse event (AE). An AE was defined as any untoward medical occurrence in a participant receiving the investigational product, regardless of its causal relationship to treatment. Participants were provided with a study card containing the research team’s contact details and were encouraged to report AEs via phone. All AEs were documented in the Case Report Form.

### 2.11. Data Analysis

Data were analyzed using IBM SPSS^®^ Statistics version 29. Between-group comparisons of mean changes in anthropometric measurements, hematological profiles, lipid profiles, and liver and renal function were performed using independent *t*-tests. Group comparisons of average weekly gastrointestinal and common cold symptom days at each time point (Month 1, 4, and 6) were conducted using the Mann–Whitney U-test. A *p*-value < 0.05 was considered statistically significant.

### 2.12. Ethical Approval and Trial Registration

Ethical approval for this study was obtained from the Human Research Ethics Committee of Universiti Sains Malaysia (JEPeM) under approval reference number USM/JEPeM/PP/23100765. The clinical trial was also registered with the National Medical Research Register of the Ministry of Health Malaysia (NMRR) under registration ID NMRR-24-02816-P0E. All procedures involving human participants were conducted in accordance with the ethical standards of the institutional research committee and with the principles outlined in the Declaration of Helsinki by the World Medical Association. Trial registration: National Medical Research Register of the Ministry of Health Malaysia (NMRR), ID NMRR-24-02816-P0E. https://nmrr.gov.my/research-directory/05254d9e-9ded-452d-a2ba-a681259294e7 (accessed on 22 March 2026).

## 3. Results

### 3.1. Participant Retention and Baseline Characteristics

At baseline, 152 participants were enrolled (76 per group). By Month 4, the placebo group decreased to 63 participants (17.1% attrition), while the probiotic group retained 66 participants (13.2% attrition). At Month 6, the placebo group further declined to 60 participants (21.1% attrition), whereas the probiotic group retained 65 participants (14.5% attrition). One participant in the placebo group was withdrawn, while all other dropouts were attributed to loss to follow-up.

Baseline demographic and anthropometric characteristics were comparable between the probiotic and placebo groups, confirming successful randomization (Table 1). The subjects in the placebo group ranged in age from 19 to 53 years, while those in the probiotic group ranged from 20 to 45 years. No subjects aged over 65 years were enrolled during the recruitment period. The mean age of participants was 27.18 ± 7.15 years in the probiotic group and 28.45 ± 9.70 years in the placebo group, with similar gender distributions. Anthropometric measures, including height, weight, and BMI, as well as cardiovascular parameters such as systolic and diastolic blood pressure and heart rate, were also comparable at baseline. Over the 24-week intervention period, no statistically significant changes were observed in these parameters in either group (Table 2).

### 3.2. Adverse Event

During the study, only one suspected adverse event was reported. A participant in the placebo group developed warm rashes with blisters on Day 9 of the intervention. The investigational product was withheld, and symptomatic treatment was administered by a medical doctor at the study site. The symptoms resolved following treatment, and the participant was withdrawn from the study. No adverse events occurred in the probiotic group throughout the trial.

### 3.3. Blood Test Results

Baseline comparisons of anthropometric and hematological parameters, as well as fasting blood sugar, showed no statistically significant differences between the placebo and probiotic groups, confirming successful randomization. Over the 24-week intervention, anthropometric measures, including body weight, BMI, and cardiovascular parameters (systolic and diastolic blood pressure, and heart rate), remained stable in both groups, with no significant between-group differences observed (Table 2).

Hematological profiles remained within normal reference ranges throughout the study. Red blood cell indices, including hemoglobin, total red blood cell count (TRBC), packed cell volume (PCV), mean corpuscular volume (MCV), and mean corpuscular hemoglobin (MCH), showed minimal fluctuations over time, with TRBC demonstrating a small but statistically significant preservation in the probiotic group compared to the placebo (Δ0–6: −0.038 vs. −0.227 million/µL, *p* = 0.015). White blood cell counts, differential proportions (neutrophils, lymphocytes, eosinophils, monocytes), platelet counts, and the inflammatory marker ESR remained within normal physiological ranges and did not differ significantly between groups.

Fasting blood sugar levels also remained stable, with no significant changes from baseline to Month 6 in either group, confirming that daily supplementation with *L. plantarum* K014 had no adverse effect on glucose metabolism. Overall, these findings indicate that the probiotic was well tolerated and did not induce clinically meaningful alterations in hematological or metabolic parameters in healthy adults.

### 3.4. Lipid Profile, Liver Function, and Kidney Function

Throughout the 24-week intervention, lipid profiles, including total cholesterol, triglycerides, HDL-cholesterol, and LDL-cholesterol, remained stable and within normal reference ranges in both the placebo and probiotic groups, with no statistically significant differences observed between groups (Table 3). These findings indicate that daily supplementation with the probiotic did not adversely affect lipid metabolism in healthy adults.

Liver function parameters were largely unchanged across groups. Hepatocellular injury markers (AST and ALT) remained within normal limits, although ALT showed a small but statistically significant decrease in the probiotic group compared to a slight increase in the placebo group (Δ0–6: −0.32 vs. 5.03 U/L, *p* = 0.035). Cholestatic function (ALP) and excretory function (total protein, albumin, globulin, and albumin/globulin ratio) remained within normal ranges. Notably, total protein, albumin, and globulin levels increased significantly in the probiotic group relative to placebo (Δ0–6: total protein 3.54 vs. 0.63 g/L, *p* = 0.014; albumin 2.94 vs. 0.97 g/L, *p* = 0.017; globulin 1.61 vs. −0.36 g/L, *p* = 0.005), indicating a possible supportive effect on protein metabolism and immune-related globulin synthesis.

Kidney function, assessed via waste filtration markers (serum creatinine, BUN, uric acid) and electrolyte balance (sodium, potassium, chloride), remained within normal reference ranges for all participants, with no clinically meaningful differences between groups. Minor fluctuations observed were statistically non-significant, confirming that *L. plantarum* K014 probiotic supplementation does not adversely affect renal function or electrolyte homeostasis in healthy adults.

### 3.5. Gastrointestinal Symptoms, Stool Consistency, and Bowel Movements

The proportion of participants reporting gastrointestinal (GI) symptoms did not differ significantly between the placebo and probiotic groups at any assessment point (Month 1, 4, and 6). Overall, the frequency of most GI symptoms was low across the study period, with no significant between-group differences observed for constipation, bloating, rectal pain, nausea, vomiting, reduced appetite, blood in stool, or tiredness at any time point (Table 4). In exploratory analyses, the diarrhea frequency per week appeared lower in the probiotic group, with statistically significant differences observed at Month 1 and Month 6 (*p* = 0.018 and *p* = 0.025, respectively).

Similarly, participants in the probiotic group experienced a statistically significant reduction in the number of days with abdominal pain at Month 6 compared to placebo (Δ0–6: 0.006 ± 0.046 vs. 0.083 ± 0.276 days/week, *p* = 0.040). Stool types associated with softer or looser stools (Type 5 and Type 7) were significantly less frequent in the probiotic group at Month 1 and Month 6 (*p* < 0.05).

### 3.6. Common Cold Symptoms and Daily Function

The proportion of participants reporting common cold symptoms did not differ significantly between the placebo and probiotic groups at any assessment point (Month 1, 4, and 6), with a general decline in symptom reporting over time in both groups (Table 5). The average number of days per week with individual common cold symptoms was low, and no significant between-group differences were observed for any symptom at any time point.

Similarly, the extent to which common cold symptoms interfered with daily activities, including focus, sleep, physical activity, social interaction, and mood control, did not differ significantly between groups throughout the study period (all *p* > 0.05). In exploratory analyses, a small but statistically significant difference was observed in the number of days participants reported interference with work/study activities at Month 1 in the probiotic group compared to placebo (0.092 ± 0.281 vs. 0.193 ± 0.327 days/week, *p* = 0.028).

## 4. Discussion

The safety evaluation of newly identified probiotic strains is essential, as probiotic effects are known to be strain-specific and cannot be extrapolated from species-level data alone. *L. plantarum* has a long history of safe use in fermented foods and dietary supplements, and numerous clinical studies have established its general safety in human populations. The present work adds to this evidence base by extending safety considerations to a previously uncharacterized strain, *L. plantarum* K014, isolated from traditional fermented vegetables, and is conducted in accordance with the Malaysian Phase I Clinical Trial Guidelines, which recommend enrollment of 50–200 participants for early-phase safety trials [12].

Anthropometric and cardiovascular parameters are commonly monitored in probiotic trials to identify unintended systemic effects. Existing literature consistently demonstrates that *L. plantarum* supplementation does not adversely influence body weight, blood pressure, or heart rate in healthy individuals [13,14,15]. These findings support the view that *L. plantarum* strains are metabolically neutral under normal physiological conditions and suitable for long-term consumption without disrupting cardiovascular or energy balance [16]. Similar findings have been reported in trials with other *L. plantarum* strains, such as P8 and 299 v, which showed no clinically relevant changes in these parameters [14,17]. Meta-analyses further confirm that probiotics generally do not significantly alter weight or blood pressure in healthy populations, supporting their safety for long-term use [18].

Hematological safety represents another critical component of probiotic assessment. Prior studies have shown that *L. plantarum* strains do not negatively affect erythropoiesis, leukocyte profiles, or inflammatory markers. Some strains have been observed to influence micronutrient bioavailability, including iron, through lactic acid production and potential effects on intestinal absorption, thereby contributing to normal hematological homeostasis without inducing pathological changes [19]. In the present study, TRBC declined significantly more in the placebo group than in the *L. plantarum* K014 group, suggesting that probiotic supplementation may attenuate the natural reduction in RBC count over time. Based on the results, white blood cell profiles, platelet counts, and ESR values remained within normal ranges in both the placebo and *L. plantarum* K014 groups throughout the study. These findings suggest that *L. plantarum* K014 does not disrupt immune cell composition, thrombopoiesis, or systemic inflammation, consistent with prior evidence of its immunological safety and non-disruptive immunomodulatory effects [14].

Metabolic safety related to glucose and lipid handling has been widely reported in probiotic research. In healthy, normoglycemic populations, probiotics typically exert minimal effects on glycemic control or lipid profiles, reflecting their role in maintaining physiological balance rather than inducing metabolic alterations [20]. In the present trial, the glucose and lipid profiles of participants remained within established physiological ranges in both groups across the study period. The patterns observed indicate that *L. plantarum* K014 did not produce discernible alterations in standard metabolic indicators under the conditions evaluated. These findings align with previous reports describing the metabolic safety of probiotics in normoglycemic and normolipidemic adult populations.

The gut–liver axis has emerged as an important pathway through which probiotics may influence hepatic health [21]. Experimental and clinical evidence suggests that *L. plantarum* strains can modulate gut microbiota composition, reduce intestinal permeability, and attenuate endotoxin translocation [22], providing biological plausibility for the hepatic safety observed across probiotic interventions. Our results confirm that *L. plantarum* K014 does not affect hepatobiliary function. These observations indicate that *L. plantarum* K014 was associated with hepatobiliary markers that remained within established physiological reference ranges under the study conditions. This pattern is consistent with prior reports describing the absence of adverse hepatic effects associated with probiotic supplementation and their interactions with bile acid–related pathways [23]. Renal safety and electrolyte balance are less frequently discussed in probiotic studies but remain important for long-term supplementation. Available evidence indicates that *L. plantarum* strains do not interfere with renal clearance mechanisms or electrolyte homeostasis in healthy individuals, contributing to the broader understanding of their suitability for daily intake [24,25,26].

Gastrointestinal tolerability is a primary determinant of probiotic acceptance and compliance. Prior research indicates that *L. plantarum* strains generally do not exacerbate gastrointestinal symptoms and may contribute to gut homeostasis through modulation of intestinal motility, bile acid metabolism, and epithelial barrier function [17,27]. However, symptomatic improvements are more consistently reported in individuals with functional gastrointestinal disorders than in healthy populations with low baseline symptom burden [28]. Probiotics have been examined for their potential to interact with host immune processes, including mucosal defense mechanisms, cytokine signaling pathways, and epithelial barrier dynamics. In healthy adults, these effects may remain subclinical and not translate into overt changes in infection-related symptoms, highlighting the need for immunological biomarkers to capture subtle probiotic–host interactions [29,30].

Several limitations inherent to probiotic safety studies should be acknowledged. Single-center designs and homogeneous study populations may limit generalizability, and reliance on self-reported outcomes may introduce reporting bias. Future studies should include more diverse populations to better evaluate safety across different demographic groups. In addition, this study involved multiple statistical comparisons, which increases the possibility of type I error and necessitates cautious interpretation of nominally significant findings. The small absolute differences observed in parameters such as TRBC, liver injury, and synthetic function markers (ALT, total protein, albumin, globulin), abdominal pain scores, stool type frequencies, total diarrhea episodes, and work/study interruption have limited clinical relevance and should be interpreted within this context. At present, no formally published preclinical safety data on *L. plantarum* K014 are available. Therefore, future studies will aim to systematically report these data to enhance strain-specific safety characterization and transparency. Furthermore, safety-focused trials may not include mechanistic endpoints such as microbiome profiling or immune biomarkers, which could provide deeper insight into host–microbe interactions. Existing evidence supports *L. plantarum* as a safe probiotic species, and the evaluation of strain K014 contributes to the growing body of strain-specific safety data. These findings provide a rationale for advancing *L. plantarum* K014 into future efficacy-oriented clinical trials and potential application in functional foods and nutraceutical formulations.

## 5. Conclusions

In conclusion, this study demonstrates that daily supplementation with *Lactiplantibacillus plantarum* K014 over a six-month period was safe and well-tolerated in healthy adults. No adverse events were reported in the probiotic group, and retention rates were higher than in the placebo group, suggesting acceptable compliance. Anthropometric, hemodynamic, and hematological parameters remained stable throughout the intervention, with no clinically relevant abnormalities observed. Markers of liver and kidney function, as well as electrolyte levels, remained within normal reference ranges. Gastrointestinal tolerability was maintained, with no evidence of symptom exacerbation, while immune-related outcomes and daily functioning were not adversely affected. Although some parameters showed minor between-group differences, these findings should be interpreted cautiously. Overall, the results support the safety of *L. plantarum* K014 for long-term consumption in healthy individuals; however, further adequately powered studies are warranted to determine whether this strain confers clinically meaningful health benefits.

## Figures and Tables

**Table 1:** Baseline comparison of demographic and anthropometric characteristics between the two groups.

	Placebo	Probiotic	*p* Value
Age (year)	28.45 ± 9.70	27.18 ± 7.15	0.363 ^a^
Gender			0.237 ^b^
Male, *n* (%)	31 (40.8%)	24 (31.5%)	
Female, *n* (%)	45 (59.2%)	52 (68.5%)	
Height (cm)	162.0 ± 9.1	161.7 ± 8.2	0.844 ^a^
Body weight (kg)	67.65 ± 19.97	63.97 ± 14.83	0.200 ^a^
Body mass index (kgm^−2^)	25.65 ± 6.74	24.46 ± 5.27	0.228 ^a^
Systolic blood pressure (mmHg)	120 ± 13	116 ± 10	0.064 ^a^
Diastolic blood pressure (mmHg)	77 ± 9	77 ± 9	0.663 ^a^
Heart rate (beats per minute)	80 ± 12	77 ± 13	0.181 ^a^

Data are expressed as mean ± SD. ^a^ Independent *t*-test; ^b^ chi-squared test.

**Table 2:** Comparison of mean differences in anthropometric characteristics, hematology profile, and fasting blood sugar between the two groups.

	Mean Differences at Each Time Point							Changes from Baseline to Month 6 (Δ0–6)		
	Baseline	Month 4	Month 6	Baseline	Month 4	Month 6	Normal Range	Placebo ^a^	Probiotic ^a^	*p* Value ^a^
	Placebo			Probiotic						
Anthropometric Characteristics										
Body weight (kg)	67.65 ± 19.97	68.31 ± 21.33	67.90 ± 20.95	63.97 ± 14.83	62.68 ± 12.27	63.49 ± 13.06	**-**	0.447 ± 2.095	0.318 ± 3.639	0.812
BMI (kgm^−2^)	25.65 ± 6.74	25.74 ± 7.03	25.54 ± 6.90	24.46 ± 5.28	24.22 ± 4.84	24.33 ± 4.93	18.5–24.9	0.140 ± 0.793	0.143 ± 1.280	0.987
SBP (mmHg)	120 ± 13	114 ± 13	115 ± 15	116 ± 10	112 ± 11	112 ± 10	<120	−4.54 ± 13.99	−4.11 ± 9.99	0.844
DBP (mmHg)	77 ± 9	73 ± 9	73 ± 9	77 ± 9	73 ± 8	71 ± 8	<80	−3.42 ± 8.48	−4.20 ± 8.66	0.615
Heart rate (per minute)	80 ± 12	83 ± 12	81 ± 13	77 ± 13	82 ± 10	80 ± 13	60–100	0.72 ± 15.47	2.98 ± 12.80	0.954
Hematology Profile—Red Blood Cell Indices										
Hgb (g/dL)	13.96 ± 1.56	14.03 ± 1.48	13.64 ± 1.56	13.68 ± 1.64	13.63 ± 1.55	13.56 ± 1.55	M: 13.5–18 F: 12–15	−0.255 ± 1.296	−0.045 ± 1.136	0.335
TRBC (million/µL)	5.23 ± 0.61	5.17 ± 0.57	5.00 ± 0.61	5.06 ± 0.58	5.05 ± 0.63	5.01 ± 0.62	M: 4.5–6.2 F: 4.0–5.5	−0.227 ± 0.421	−0.038 ± 0.433	0.015
PCV (%)	44.68 ± 4.36	45.13 ± 4.46	43.03 ± 4.67	43.96 ± 4.73	44.12 ± 4.22	42.29 ± 6.70	M: 38–50 F: 34–44	−1.47 ± 4.98	−1.31 ± 6.62	0.879
MCV (fL)	86.87 ± 7.65	88.11 ± 7.57	86.47 ± 7.88	85.89 ± 8.15	88.29 ± 8.72	85.74 ± 11.55	78–99	−0.17 ± 5.38	0.67 ± 5.54	0.395
MCH (pg)	27.53 ± 2.74	27.52 ± 2.65	27.58 ± 2.73	27.04 ± 2.92	27.41 ± 3.23	27.64 ± 3.29	27–32	0.07 ± 1.55	0.63 ± 2.19	0.102
Hematology Profile—White Blood Cell Profile										
TWBC (cells/µL)	7186 ± 1374	7177 ± 1375	7140 ± 1536	6953 ± 1397	6716 ± 1635	6648 ± 1510	M: 5000–10,000 F: 4500–11,000	−101 ± 1632	−325 ± 1759	0.480
Neutrophil (%)	60.39 ± 8.29	60.54 ± 9.08	62.08 ± 7.75	59.68 ± 8.95	58.42 ± 9.54	60.44 ± 8.26	40–75	1.73 ± 10.40	0.11 ± 11.33	0.404
Lymphocyte (%)	29.74 ± 8.01	29.43 ± 8.39	28.12 ± 7.78	30.38 ± 8.81	31.65 ± 9.22	29.64 ± 8.10	15–45	−1.70 ± 10.07	−0.11 ± 11.12	0.402
Eosinophil (%)	4.91 ± 0.50	4.57 ± 0.88	3.92 ± 1.29	4.95 ± 0.36	4.59 ± 0.98	4.17 ± 1.37	0–6	−0.97 ± 1.39	−0.79 ± 1.41	0.475
Monocyte (%)	4.96 ± 0.34	5.38 ± 1.39	6.08 ± 1.29	4.99 ± 0.26	5.33 ± 1.24	5.76 ± 1.38	0–10	1.13 ± 1.32	0.79 ± 1.41	0.159
Hematology Profile—Platelet Count										
Platelet count (per mcL)	269,526 ± 62,522	278,269 ± 66,059	267,650 ± 54,339	277,723 ± 71,589	289,939 ± 63,661	279,878 ± 56,077	150,000–450,000	800 ± 62,677	−182 ± 61,314	0.929
Hematology Profile—Inflammatory Marker										
ESR (mm/hour)	3.09 ± 0.79	2.94 ± 0.88	3.13 ± 0.62	3.36 ± 1.36	2.73 ± 0.85	3.09 ± 0.66	M: 0–20 F: 0–30	0.00 ± 1.14	−0.38 ± 1.53	0.438
Fasting Blood Sugar										
FBS (mmol/L)	4.62 ± 0.79	4.64 ± 0.69	4.56 ± 1.14	4.45 ± 0.40	4.46 ± 0.49	4.33 ± 0.37	3.9–5.6	−0.065 ± 0.667	−0.108 ± 0.440	0.671

Data are expressed as mean ± SD. ^a^ An independent *t*-test was used to compare the mean differences in changes in the profile between the two groups. BMI: body mass index; SBP: systolic blood pressure; DBP: diastolic blood pressure; Hgb: hemoglobin; TRBC: total red blood cell; PCV: packed cell volume; MCV: mean corpuscular volume; MCH: mean corpuscular hemoglobin; TWBC: total white blood cell; ESR: erythrocyte sedimentation rate; FBS: fasting blood sugar; M: male; F: female.

**Table 3:** Comparison of mean differences in lipid profile, liver function, and kidney function between the two groups.

	Mean at Each Time Point							Changes from Baseline to Month 6 (Δ0–6)		
	Baseline	Month 4	Month 6	Baseline	Month 4	Month 6	Normal Range	Placebo ^a^	Probiotic ^a^	*p* Value ^a^
	Placebo			Probiotic						
Lipid Profile										
Total cholesterol (mmol/L)	5.17 ± 0.81	5.26 ± 0.97	5.47 ± 1.01	5.04 ± 0.95	5.09 ± 0.85	5.37 ± 0.72	<5.2	0.365 ± 1.023	0.425 ± 0.890	0.737
Triglyceride (mmol/L)	1.09 ± 0.65	1.01 ± 0.58	1.15 ± 0.72	0.95 ± 0.54	0.95 ± 0.47	1.01 ± 0.38	<1.7	0.177 ± 0.717	0.054 ± 0.337	0.511
HDL-cholesterol (mmol/L)	1.65 ± 0.36	1.71 ± 0.37	1.62 ± 0.3 T7	1.69 ± 0.37	1.80 ± 0.32	1.66 ± 0.32	>1.04	−0.052 ± 0.396	−0.026 ± 0.393	0.846
LDL-cholesterol (mmol/L)	3.06 ± 1.03	3.28 ± 1.25	3.47 ± 1.32	2.95 ± 1.12	3.11 ± 1.10	3.30 ± 1.05	Optimal: <2.6; Near optimal: 2.6–3.3	0.487 ± 1.341	0.498 ± 1.185	0.716
Liver Function (Hepatocellular Injury Markers)										
AST (U/L)	20.18 ± 7.69	22.76 ± 10.90	20.25 ± 8.32	19.25 ± 9.50	22.79 ± 9.55	18.61 ± 9.74	5–40	0.32 ± 8.73	−0.09 ± 11.89	0.828
ALT (U/L)	19.16 ± 15.10	20.70 ± 17.17	23.42 ± 16.17	18.96 ± 14.66	20.30 ± 18.19	17.71 ± 7.79	7–56	5.03 ± 14.58	−0.32 ± 13.62	0.035
Liver Function—Cholestatic Marker										
ALP (U/L)	68.87 ± 18.77	73.24 ± 21.98	78.95 ± 20.59	70.89 ± 20.61	72.48 ± 22.05	78.80 ± 23.00	40–129	7.85 ± 24.13	7.18 ± 29.29	0.890
Liver Function—Excretory Function Marker										
Total bilirubin (µmol/L)	9.28 ± 4.99	10.40 ± 4.77	9.00 ± 4.56	8.89 ± 4.80	9.03 ± 3.80	8.89 ± 4.35	3–24	−0.65 ± 5.58	−0.31 ± 4.74	0.712
Liver Function—Synthetic Function Marker										
Total protein (g/L)	76.97 ± 6.07	79.16 ± 3.95	77.68 ± 4.42	76.01 ± 5.27	78.02 ± 3.17	79.42 ± 3.86	63–83	0.63 ± 6.91	3.54 ± 6.15	0.014
Albumin (g/L)	44.38 ± 3.35	45.25 ± 2.69	45.60 ± 3.40	44.25 ± 3.16	45.12 ± 2.31	47.26 ± 3.93	35–50	0.97 ± 4.44	2.94 ± 4.65	0.017
Globulin (g/L)	32.80 ± 3.76	33.92 ± 3.25	32.37 ± 3.08	31.76 ± 3.31	32.97 ± 2.82	33.20 ± 3.16	22–40	−0.36 ± 3.88	1.61 ± 3.81	0.005
Albumin/ Globulin ratio	1.37 ± 0.181	1.35 ± 0.164	1.40 ± 0.168	1.39 ± 0.144	1.38 ± 0.165	1.42 ± 0.167	1.0–2.2	0.02 ± 0.20	0.02 ± 0.17	0.902
Kidney Function—Waste Filtration Markers										
Serum creatinine (µmol/L)	67.95 ± 15.85	74.59 ± 14.80	75.67 ± 17.74	68.38 ± 16.04	72.55 ± 16.26	75.63 ± 15.39	60–110	7.93 ± 14.85	9.34 ± 13.61	0.592
Blood urea nitrogen (mmol/L)	4.02 ± 1.00	4.53 ± 1.05	4.40 ± 1.30	3.81 ± 1.09	4.46 ± 1.25	4.30 ± 1.47	1.7–8.3	0.415 ± 1.237	0.610 ± 1.269	0.394
Uric acid (µmol/L)	321.6 ± 104.4	318.6 ± 96.7	328.1 ± 93.5	296.6 ± 80.4	309.6 ± 88.6	317.3 ± 83.2	M: 202–416; F: 142–339	9.4 ± 81.8	20.8 ± 59.7	0.391
Kidney Function—Electrolyte Balance										
Serum sodium (mmol/L)	143.38 ± 3.29	142.60 ± 2.83	142.15 ± 3.17	142.21 ± 3.33	143.03 ± 2.92	141.97 ± 3.27	135–145	−1.07 ± 4.03	−0.30 ± 4.49	0.324
Serum potassium (mmol/L)	4.23 ± 0.23	4.18 ± 0.15	4.13 ± 0.23	4.17 ± 0.16	4.18 ± 0.16	4.17 ± 0.11	3.5–5.0	−0.086 ± 0.290	−0.014 ± 0.196	0.113
Serum chloride (mmol/L)	101.28 ± 2.72	101.22 ± 2.53	100.69 ± 2.24	101.03 ± 2.79	100.75 ± 2.15	100.63 ± 2.12	98–106	−2.42 ± 13.91	−0.41 ± 3.42	0.269

Data are expressed as mean ± SD. ^a^ An independent *t*-test was used to compare the mean differences in changes in the profile between the two groups. HDL-C: high-density lipoprotein cholesterol; LDL-cholesterol: low-density lipoprotein cholesterol; AST: aspartate aminotransferase; ALT: alanine aminotransferase; ALP: alkaline phosphatase; M: male; F: female.

**Table 4:** Average weekly GIT symptom days, stool, and diarrhea frequency per participant.

	Month 1			Month 4			Month 6		
	Placebo	Probiotic	*p* Value	Placebo	Probiotic	*p* Value	Placebo	Probiotic	*p* Value
Experience of GIT symptoms (Per Week) ^a^									
Yes (%)	36.4	44.6	0.832	16.5	21.5	0.320	9.5	12.6	0.462
Number of Days with GIT Symptoms (Per Week) ^b^									
Constipation	0.343 ± 0.628	0.262 ± 0.463	0.904	0.144 ± 0.329	0.089 ± 0.413	0.383	0.165 ± 0.443	0.091 ± 0.402	0.252
Bloating	0.377 ± 0.697	0.509 ± 0.897	0.833	0.215 ± 0.478	0.173 ± 0.601	0.434	0.1075 ± 0.332	0.138 ± 0.625	0.811
Diarrhea	0.422 ± 0.654	0.315 ± 0.638	0.101	0.234 ± 0.476	0.120 ± 0.339	0.272	0.098 ± 0.237	0.067 ± 0.306	0.102
Rectal Pain	0.073 ± 0.229	0.092 ± 0.265	0.927	0.061 ± 0.252	0.007 ± 0.052	0.193	0.045 ± 0.163	0.030 ± 0.164	0.441
Abdominal Pain	0.218 ± 0.407	0.250 ± 0.548	0.526	0.090 ± 0.212	0.055 ± 0.189	0.717	0.083 ± 00.276	0.006 ± 0.046	0.040
Nausea	0.059 ± 0.204	0.119 ± 0.291	0.155	0.084 ± 0.249	0.065 ± 0.418	0.066	0.045 ± 0.163	0.065 ± 0.490	0.240
Vomiting	0.007 ± 0.053	0.029 ± 0.121	0.924	0.063 ± 0.282	0.029 ± 0.210	0.444	0.023 ± 0.120	0.058 ± 0.391	0.800
Reduce Appetite	0.093 ± 0.284	0.119 ± 0.241	0.305	0.147 ± 0.347	0.029 ± 0.125	0.087	0.038 ± 0.174	0.024 ± 0.159	0.448
Blood in Stool	0.015 ± 0.105	0.006 ± 0.047	0.885	0.016 ± 0.077	0.000 ± 0.000	0.107	0.015 ± 0.106	0.000 ± 0.000	0.256
Tiredness	0.308 ± 0.737	0.319 ± 0.710	0.300	0.166 ± 0.488	0.007 ± 0.052	0.136	0.095 ± 0.309	0.058 ± 0.238	0.275
Number of Days with Experience Respective Stool Consistency (Per Week) ^b^									
Stool Type 1	0.292 ± 0.675	0.224 ± 0.443	0.991	0.245 ± 0.579	0.230 ± 0.527	0.888	0.405 ± 0.855	0.269 ± 0.644	0.554
Stool Type 2	0.594 ± 0.805	0.780 ± 1.174	0.894	0.832 ± 1.174	0.970 ± 1.349	0.812	0.970 ± 1.636	1.030 ± 1.608	0.687
Stool Type 3	2.240 ± 1.753	2.526 ± 1.758	0.497	2.745 ± 1.800	2.515 ± 2.033	0.505	2.440 ± 1.979	2.567 ± 2.120	0.987
Stool Type 4	1.661 ± 1.423	1.610 ± 1.596	0.960	1.674 ± 1.720	1.605 ± 2.065	0.144	1.935 ± 2.214	1.859 ± 2.367	0.779
Stool Type 5	0.646 ± 1.004	0.276 ± 0.643	0.012	0.147 ± 0.336	0.110 ± 0.379	0.217	0.160 ± 0.334	0.131 ± 0.503	0.023
Stool Type 6	0.286 ± 0.508	0.121 ± 0.308	0.060	0.065 ± 0.271	0.130 ± 0.709	0.508	0.055 ± 0.170	0.086 ± 0.482	0.273
Stool Type 7	0.172 ± 0.365	0.039 ± 0.131	0.052	0.049 ± 0.261	0.025 ± 0.104	0.725	0.025 ± 0.116	0.000 ± 0.000	0.045
Number of Days with Experience Respective Stool Color (Per Week) ^b^									
Brown	5.172 ± 1.531	5.178 ± 1.893	0.946	5.283 ± 1.603	5.190 ± 1.697	0.440	5.555 ± 1.627	5.429 ± 1.890	0.788
Yellow	0.484 ± 0.913	0.301 ± 0.774	0.807	0.400 ± 0.848	0.375 ± 0.860	0.292	0.315 ± 0.886	0.358 ± 1.186	0.918
Black	0.313 ± 0.721	0.127 ± 0.329	0.407	0.156 ± 0.501	0.145 ± 0.474	0.928	0.060 ± 0.245	0.175 ± 00.648	0.270
Green	0.021 ± 0.113	0.021 ± 0.116	0.789	0.044 ± 0.298	0.000 ± 0.000	0.259	0.020 ± 0.111	0.000 ± 0.000	0.406
Stool Frequency (Per Week) ^b^									
Total Bowel Movements	8.630 ± 4.964	7.787 ± 4.478	0.710	7.936 ± 3.702	8.005 ± 5.736	0.083	8.405 ± 4.603	8.220 ± 5.147	0.987
Total Diarrhea Frequency	1.178 ± 1.495	0.487 ± 0.879	0.018	0.505 ± 0.997	0.293 ± 0.885	0.068	0.440 ± 1.030	0.239 ± 0.758	0.025

Data are expressed as mean ± SD. *p* values denote between-group comparisons. ^a^ *p* value obtained via chi-squared test; ^b^ *p* value obtained via Mann–Whitney U-test.

**Table 5:** Average weekly common cold symptom days, stool, and diarrhea frequency per participant.

	Month 1			Month 4			Month 6		
	Placebo	Probiotic	*p* Value	Placebo	Probiotic	*p* Value	Placebo	Probiotic	*p* Value
Experience of Common Cold Symptoms (Per Week) ^a^									
Yes (%)	20.4	24.6	0.816	13.1	16.9	0.422	11.3	14.7	0.743
Number of Days with Common Cold Symptoms (Per Week) ^b^									
Fever	0.214 ± 0.471	0.123 ± 0.336	0.543	0.089 ± 0.288	0.040 ± 0.160	0.791	0.120 ± 0.346	0.106 ± 0.335	0.431
Blocked Nose	0.446 ± 0.681	0.341 ± 0.869	0.210	0.227 ± 0.535	0.092 ± 0.261	0.319	0.112 ± 0.287	0.223 ± 0.900	0.943
Sneezing	0.518 ± 0.739	0.492 ± 0.996	0.415	0.283 ± 0.703	0.094 ± 0.249	0.514	0.138 ± 0.336	0.213 ± 0.543	0.914
Sore Throat	0.204 ± 0.458	0.181 ± 0.377	0.874	0.139 ± 0.342	0.066 ± 0.224	0.174	0.088 ± 0.271	0.162 ± 0.404	0.667
Throat Itchiness	0.184 ± 0.435	0.222 ± 0.622	0.908	0.181 ± 0.593	0.080 ± 0.243	0.567	0.060 ± 0.233	0.258 ± 0.935	0.154
Cough	0.345 ± 6.535	0.283 ± 0.829	0.498	0.259 ± 0.611	0.099 ± 0.317	0.226	0.058 ± 0.252	0.269 ± 0.947	0.177
Hoarseness of Voice	0.163 ± 0.469	0.123 ± 0.367	0.987	00.097 ± 0.393	0.052 ± 0.195	0.602	0.074 ± 0.261	0.171 ± 0.418	0.458
Headache	0.265 ± 0.458	0.235 ± 0.433	0.611	0.108 ± 0.237	0.019 ± 0.083	0.079	0.065 ± 0.219	0.092 ± 0.260	0.851
Shortness of Breath	0.105 ± 0.288	0.070 ± 0.210	0.830	0.017 ± 0.078	0.022 ± 0.116	0.458	0.000 ± 0.000	0.023 ± 0.112	0.474
Tiredness	0.543 ± 0.824	0.431 ± 0.690	0.756	0.119 ± 0.299	0.0443 ± 0.142	0.246	0.143 ± 0.330	0.121 ± 0.355	0.294
Severity of Common Cold Symptoms Interfered with Ability (Per Week) ^b^									
Focus	0.167 ± 0.298	0.096 ± 0.237	0.131	0.071 ± 0.209	0.024 ± 0.089	0.853	0.075 ± 0.184	0.073 ± 0.196	0.846
Sleep	0.278 ± 0.477	0.150 ± 0.257	0.751	0.0598 ± 0.176	0.024 ± 0.101	0.41	0.056 ± 0.171	0.058 ± 0.158	0.252
Breath	0.136 ± 0.272	0.082 ± 0.223	0.793	0.065 ± 0.178	0.033 ± 0.120	0.8859	0.040 ± 0.105	0.065 ± 0.189	0.215
Walk, Climb Stairs, Exercise	0.117 ± 0.255	0.105 ± 0.274	0.444	0.005 ± 0.037	0.009 ± 0.048	0.38	0.035 ± 0.113	0.039 ± 0.127	1.000
Accomplish Daily Living Activity	0.122 ± 0.260	0.077 ± 0.250	0.242	0.027 ± 0.151	0.009 ± 0.048	0.853	0.045 ± 0.120	0.051 ± 0.168	0.383
Work/Study	0.193 ± 0.327	0.092 ± 0.281	0.028	0.043 ± 0.121	0.024 ± 0.089	0.38	0.055 ± 0.145	0.046 ± 0.146	0.383
Interact with Others	0.085 ± 0.217	0.054 ± 0.195	0.748	0.033 ± 0.126	0.014 ± 0.058	0.853	0.050 ± 0.134	0.066 ± 0.190	0.215
Control Daily Mood	0.112 ± 0.254	0.061 ± 0.202	0.71	0.061 ± 0.221	0.014 ± 0.058	0.205	0.060 ± 0.164	0.035 ± 0.124	1.000

Data are expressed as mean ± SD. *p* values denote between-group comparisons. ^a^ *p* value obtained via the chi-squared test; ^b^ *p* value obtained via the Mann–Whitney U-test.

## Data Availability

Data is available upon request from the corresponding authors.

## Supplementary Materials

The following supporting information can be downloaded at: https://www.mdpi.com/article/10.3390/nu18091406/s1, Table S1: CONSORT 2010 checklist of information to include when reporting a randomised trial *.
